# Treating Chronic Rhinitis and Turbinate Hypertrophy Without Surgery: The Effectiveness of Silver Nitrate Cauterization

**DOI:** 10.7759/cureus.35758

**Published:** 2023-03-04

**Authors:** Oguz Guvenmez, Anara Keneshovna Zhanbaeva, Huseyin Keskin, Adylbek Zhanbaev

**Affiliations:** 1 Otorhinolaryngology, Osh State University, Osh, KGZ; 2 Clinical Pharmacology, Osh State University, Osh, KGZ; 3 Otorhinolaryngology, Özel Mersin Su Hastanesi, Mersin, TUR; 4 General Surgery, Osh State University, Osh, KGZ

**Keywords:** chronic rhinitis, allergic rhinitis, nasal obstruction treatment, chronic rhinitis treatment, turbinate hypertrophy, silver nitrate cauterization

## Abstract

Introduction: Chronic rhinitis and chronic turbinate hypertrophy are conditions that affect the quality of life of individuals. The conchas, or the three half-crescent-shaped structures in the nasal cavity, play a crucial role in the respiratory system by filtering and humidifying the air we breathe. The growth of the conchas outside of normal physiological conditions can lead to conditions such as hyperplasia of the inferior turbinate and hypertrophy of the inferior turbinate.

Methods: The study was conducted between June 2020 and December 2022 and aimed to evaluate the effect of silver nitrate cauterization on patients with chronic rhinitis and chronic turbinate hypertrophy. A total of 638 patients and 520 controls with nasal obstruction were included in the study and underwent silver nitrate cauterization without the need for surgical intervention. The treatment was performed under local anesthesia and involved the application of silver nitrate on the anterior one-centimeter part of the medial surface of both inferior turbinates. Patients were instructed to use an isotonic solution nasal spray and take an antihistamine pill for seven days.

Results: A significant improvement was observed in the overall inspiratory function without the need for nasal decongestant sprays or surgery in all patients after one and three months. There was also a significant improvement noted three months after the silver nitrate cauterization in the Nasal Obstruction Symptom Evaluation (NOSE) scores, which measure the level of nasal obstruction.

Conclusion: Silver nitrate cauterization showed to be an effective treatment for patients with turbinate hypertrophy, leading to an improvement in overall inspiratory function and reducing the severity of nasal obstruction. This treatment can serve as an alternative to surgical intervention for these conditions.

## Introduction

Chronic rhinitis and chronic turbinate hypertrophy are prevalent conditions that have a significant impact on an individual's quality of life. The nasal cavity is made up of three conchas (the lower, middle, and upper) located on the side walls of the nasal cavity [1]. These organ-like structures, shaped like a half crescent, play a crucial role in the respiratory system by filtering, humidifying, and heating the air we breathe before it reaches the lower airways and lungs [2].

The conchas perform their functions by providing a large surface area for the exchange of heat and moisture with the inspired air. The lower conchas direct the airflow, allowing for maximum contact with the mucous layer, which acts as a filter, trapping any particles or allergens that may be present in the air. The middle turbinates primarily protect the sinus drainage pathways and help regulate air circulation by directing the airflow away from these passages, while the upper turbinates protect the olfactory region, responsible for our sense of smell [3].

The conchas contain dense vascular networks that are capable of rapidly adapting to changes in environmental conditions. This means that the size of the conchas can change depending on outdoor air conditions, making the nose a dynamic airway that adapts to external conditions [4].

Hyperplasia of the inferior turbinate (HIT) is a common cause of nasal obstruction and can result in chronic nasal discharge, mouth breathing, open-mouth sleep, sleep apnea, and craniofacial disorders [5]. Causes of HIT include allergic and non-allergic rhinitis, exposure to pollutants and chemicals, active or passive smoking, and various drugs. HIT is a widespread cause of nasal blockage in populations. HIT-induced chronic nasal obstruction has a negative impact on individuals' quality of life. Chronic nasal discharge can result in pathologies such as continuous mouth breathing, open-mouth sleeping, sleep apnea, and craniofacial disorders [6]. Allergic and non-allergic rhinitis are the primary causes of HIT [7]. Topical and systemic antihistamines and intranasal steroids are prescribed as treatments [8]. However, the prolonged use of these drugs and their limited effectiveness are major concerns.

Currently, surgical procedures such as turbinectomy, submucosal resection, and radiofrequency cautery are performed on patients who do not respond to medical treatments for chronic rhinitis and chronic turbinate hypertrophy [9-11]. The objective of this study is to demonstrate that lower turbinate hypertrophy can be treated without the need for surgical intervention.

## Materials and methods

The present study was conducted between June 2020 and December 2022 at Mersin Private Su Hospital and aimed to evaluate the effect of silver nitrate cauterization on patients with hypertrophic turbinates. The study was approved by the Mersin University Ethics Committee (approval number: 01.03.2023/125). A total of 638 patients and 520 controls with nasal obstruction who were admitted to the otorhinolaryngology outpatient clinic of the hospital were included in the study. Patients with lower turbinate hypertrophy who wanted medical treatment were accepted as the control group. In other words, patients who did not agree to undergo any invasive procedure were considered the control group.

Patients' complaints and medical histories were recorded, and those with adenoid vegetation, grossly deviated nasal septum, nasal polyps, or other causes of nasal obstruction were excluded from the study. Additionally, patients without pre-treatment Nasal Obstruction Symptom Evaluation (NOSE) scores or without two post-treatment NOSE scores were also excluded from the study.

All patients underwent silver nitrate cauterization without the need for surgical reduction of the inferior turbinate. The treatment was carried out by a specialist otolaryngologist and was performed under local anesthesia. To achieve local anesthesia, pre-prepared xylocaine-impregnated cotton strips were utilized. These strips were inserted into the patient's nostrils, and a decongestant spray containing oxymetazoline was also applied. A 0-degree endoscope was then inserted.

The next step involved the application of a previously prepared solid form of silver nitrate rod on the anterior one-centimeter part of the medial surface of both inferior turbinates until a white discoloration was observed in the mucosa covering the turbinates.

The treatment was performed under local anesthesia and involved the application of silver nitrate on the anterior one-centimeter part of the medial surface of both inferior turbinates. Patients were instructed to use an isotonic solution nasal spray and take an antihistamine pill for seven days.

Patients were instructed to avoid picking their nose and to use isotonic solution nasal spray five times a day for seven days. It was also recommended that they take an antihistamine pill (fexofenadine 180 mg) once a day for the following seven days to prevent sneezing or runny nose. Even if there was no complaint of pain, a nonsteroidal anti-inflammatory drug (diclofenac potassium 50 mg) was prescribed.

## Results

The study included a total of 638 patients and 520 controls. The demographic data are presented in Table 1. The age range of the patients was between 15 and 65 years. The first group consisted of 638 patients and the second group consisted of 520 patients. The gender distribution of the first group was 342 (53.6%) females and 296 (46.4%) males, while the second group consisted of 284 (54.6%) females and 284 (54.6%) males. The mean age was 38.7 ± 15.1 years in group I and 39.1 ± 14.9 years in group II.

**Table 1:** Demographic variables and clinical characteristics of the sample A: Independent sample t-test was used. B: Chi-square test was used.

		Group I		Group II		p^A^
		Mean ± SD/N (%)	Median	Mean ± SD/N (%)	Median	
Age		38.7 ± 15.1	36.0	39.1 ± 14.9	37.0	0.722
						p^B^
Gender	Male	296/46.4%		236/45.4%		0.128
	Female	342/53.6%		284/54.6%		0.256

According to the standard protocol of the ear, nose, and throat clinic, a NOSE scale questionnaire was administered to the patients during their first visit and repeated at one and three months intervals after the treatment. The NOSE scale consists of five questions that assess the level of nasal obstruction and each symptom is evaluated on a Likert scale ranging from 0 (not a problem) to 4 (severe problem) (Table 2). The total NOSE score ranges from 0 to 100, with higher scores indicating greater severity of nasal obstruction. The scores were classified into mild (5-25), moderate (30-50), severe (55-75), or extreme (80-100) severity ranges [11].

**Table 2:** NOSE scale NOSE: Nasal Obstruction Symptom Evaluation.

NOSE instrument	Not a problem	Very mild problem	Moderate problem	Fairly bad problem	Severe problem
Nasal stuffiness	0	1	2	3	4
Nasal blockage or obstruction	0	1	2	3	4
Trouble breathing through my nose	0	1	2	3	4
Trouble sleeping	0	1	2	3	4
Unable to get enough air through my nose during exercise or exertion	0	1	2	3	4

There was no significant difference in the mean NOSE scores of all patients before treatment (p = 0.613), as shown in Table 3. However, there was a significant decrease in NOSE scores compared to the pre-treatment scores in group I one month after treatment (p = 0.014). In contrast, the mean NOSE score of group I was significantly lower than the mean NOSE score of group II in the first and third months after treatment (p = 0.001).

**Table 3:** The comparison of group I and group II NOSE scores A: Independent sample t-test was used. Paired sample t-test was used. NOSE: Nasal Obstruction Symptom Evaluation.

	NOSE	Group I	Group II	p^A^
		Mean ± SD	Mean ± SD	
	Before treatment	65.1 ± 4.3	64.9 ± 4.5	0.613
	After treatment (1 month)	34.2 ± 3.4	63.8 ± 5.2	0.014
	After treatment (3 months)	10.5 ± 2.8	64.4 ± 5.1	0.001

Before the treatment, the average NOSE score for all patients was 62.0 ± 14.1, indicating moderate to severe nasal congestion. The pre-treatment NOSE score was higher for females compared to males. However, both males and females showed statistically significant improvement in their NOSE scores after receiving the nasal topical treatment, with p-values less than 0.01 for both groups as determined by the paired t-test. After the treatment, the average NOSE score was 20 ± 9.3.

**Figure 1:**
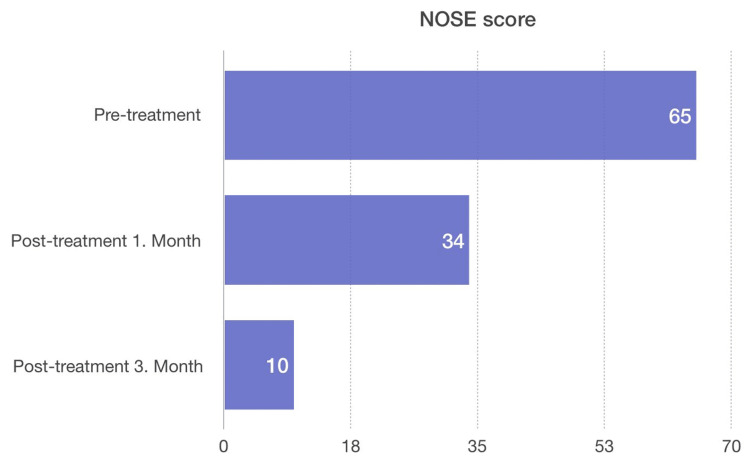
Comparison of NOSE scores between pre-treatment and post-treatment NOSE: Nasal Obstruction Symptom Evaluation.

As shown in Figure 1, when both groups were compared, it was observed that the NOSE score was significantly lower in favor of group I when the pre-treatment and post-treatment third-month NOSE scores were compared.

Statistical analysis

In this study, a range of statistical techniques was applied to analyze the data and draw meaningful conclusions. Descriptive statistics were used to determine the standard deviation, mean, median, lowest, highest, frequency, and ratio values of the data. The distribution of the variables was assessed with the Kolmogorov-Smirnov test, while the chi-square test was used to compare categorical variables. The t-tests, both the independent sample and paired sample, were also used in the analysis. The results of the analysis were produced using SPSS 21.0 program (IBM Corp., Armonk, NY).

## Discussion

The treatment of lower turbinate hypertrophy has been explored through various techniques to date [12]. However, many of these methods have not been effective in preserving the essential functions of the nose while alleviating symptoms. Partial concha resections have been reported to reduce nasal obstruction by 41-90%, but they come with significant risks such as severe synechia, crusting, and bleeding [13]. On the other hand, submucous turbinate resection is a less invasive technique, but it still poses a 14% risk of complications such as bleeding [14]. Microdebrider-assisted submucosal resection is a recent method but has been reported to result in bleeding, synechia, and mucosal damage in 62% of cases [15]. Overall, the surgical treatments for concha hypertrophy pose both short- and long-term risks.

The use of topical long-term intranasal steroids is another method of treating lower turbinate hypertrophy [16-18]. While some studies suggest its effectiveness, the long-term use of this treatment may lead to adverse effects such as nosebleeds, nasal dryness, crusting, and septal perforation [19].

In contrast, our study showed that the silver nitrate cauterization treatment is easy to administer, effective in improving nasal breathing, cost-effective, and has fewer complications. No septal perforation was detected in control examinations performed one and three months after the treatment. Patients reported no symptoms of nasal dryness, nosebleed, or crusting.

**Figure 2:**
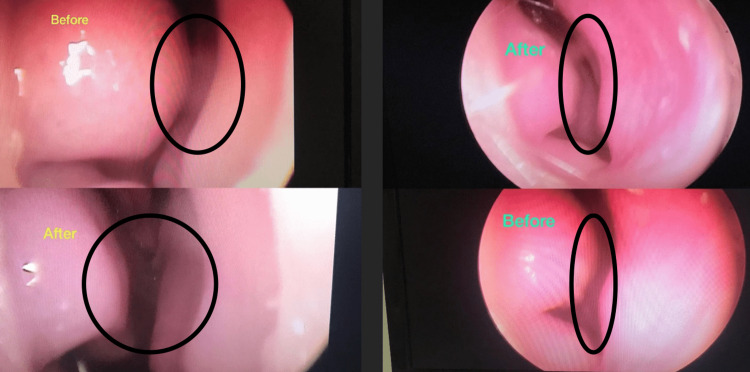
Before and after three months of silver nitrate treatment

Following the treatment with silver nitrate, a significant improvement was observed in the overall inspiratory function without the need for nasal decongestant sprays or surgery in all patients after one and three months (p = 0.001). As shown in Figure 2, there was also a significant improvement noted three months after the silver nitrate treatment.

In a clinical study conducted by Harrill et al., the authors compared the treatment efficacy of a group of patients with bilateral inferior turbinate radiofrequency plus septoplasty and a group of patients with bilateral inferior turbinate radiofrequency using the NOSE scale. They showed a significant improvement in NOSE scores for both groups. There was no statistically significant difference between the two treatment groups in terms of postoperative outcomes [20]. In this study, the patients were operated on under anesthesia in the operating room environment. In our study, silver nitrate treatment was performed in the outpatient clinic using only local anesthesia. This treatment we apply is less costly economically and more easily applicable for the patient without entering the operating room.

This study showed that turbinate hypertrophy can be easily reduced and nasal obstruction can be relieved in outpatient settings. In addition, no complications occurred. On the other hand, long-term medical treatments and surgical interventions create a serious economic burden on the society and the country. Silver nitrate treatment in the study is very low cost. In this way, it saves from an important economic burden.

The facts that the study was single-centered and evaluated by a single specialist doctor are the conditions that limit the study. In addition, this study has a limitation in that acoustic rhinomanometry was not used for the objective evaluation of nasal obstruction. Further studies combining objective and subjective evaluations are needed to evaluate the efficacy of treatment using silver nitrate in patients with nasal obstruction.

## Conclusions

Silver nitrate, with a local effect, causes destruction in the nasal mucosa and reduces the turbinate. Thus, it becomes easier to breathe through the nose without surgical complications. In conclusion, the results of this study suggest that silver nitrate cauterization is a promising, non-surgical treatment option for lower turbinate hypertrophy. For patients, this treatment is easily acceptable and can be easily repeated if symptoms recur. Side effects are minor and no premedication is required. It does not require detailed equipment or a surgical team. It is important that this treatment is disseminated in the society so that people can breathe more easily.
